# Kill or Cure

**Published:** 1859-07

**Authors:** 


### KILL OR CURE,

Neck or nothing, are favorite saws with some people, and with other some, a little more daft, there is a still more dearly hugged comforter, "so simple," that it can't do any harm, if it does no good; and armed with that philosophy, multitudes daily swallow poisons to an incredible amount, with the result of losing the last remnant of health, if not life itself. They start out on the assumed fact, that what is "simple" can't injure. If this is applied to men, we think it rather unfortunate, for there are "simple" men and women in myriads, who are doing hourly more harm to themselves, their friends, their neighbors, their children, than any arithmetic can compute; so simple in eating, in dress, in opinion, in conversation, in judgment, in conduct, that often the expression escapes themselves in reviewing the past, "what a fool I am!"

But this is a moral simplicity. The simplicity of remedial agents is the subject more immediately in hand. The people who are so marvellously fond of what they call "simple" things, start out on the unwarrantable supposition, that what is "simple" is synonymous with the fact that they are "familiar" with it. Whiskey for example, is a familiar, and we might say, a very familiar article with some people; verily it is with them an old acquaintance, a bosom friend, an inseparable companion; their testimony is uniformly that it is good for the "insides" and good for the out; that it not only never did them any harm, but always did them good; they "always felt better after taking it."

We are very well acquainted with tobacco. Look at the Virginian for example: he talks of tobacco, he dreams about it, he eats it, he smells of it; the very dollar in his pocket is redolent with its hateful fumes; it is wedged in under his finger nails, it spots his shirt bosom, it stains his vest, its juice is scattered over his pants, it cakes at the corners of his mouth, and the long streaks of colored saliva dribble from his lip, and stripe his cheeks. As he uses it more, the necessity for its use is greater, until finally he goes to sleep with a lump of it in his mouth. Next he begins to dry up, his flesh shrinks away, his face is gaunt, his body slab-like, his legs spindles, his gait is tottering and unsteady, and head and fingers and arms shake like the palsied or the agued. Next comes the wasting of the life powers; digestion ceases, appetite fails, the nervous energies are exhausted, and dullness and stupor and the sleep of death come on.

Coffee and tea are very "simple," very familiar things, and have been used for a life time by multitudes without any noticeably injurious effects which could be fairly and conclusively attributed to them; but "simple" as they are, their injudicious use has made many an one miserably nervous and dyspeptic for life. "Simple" would it seem to rub a little candle grease on a trifling pimple, and yet death followed from the poisonous corrosion of the brass candlestick.

Then again, what may be "simple" and safe for one, may not be simple and safe for another. The tired donkey found his oppressive load of salt lightened, and himself greatly refreshed by swimming a swollen stream; but his brother donkey, loaded with a huge sack of wool, was delighted at the instant relief afforded to him by the same means, but it was a transcient and deceitful remedy, for no sooner did he begin to emerge from the stream, than the increased weight of wool and water crushed him hopelessly.

A newspaper writer, as green as the grass he treads upon, recommends what he considers a very "simple" remedy—Ice. Hear him:—

"Attacked with pneumonia, salivated, broken down in constitution, subject to hæmorrhages from the lungs, digestion totally deranged, and rheumatic neuralgia, he tried in vain the remedies prescribed by American physicians, the effects of foreign travel, the most rigid diet, and the most careful and systematic habits of life. The most learned physicians of London, Paris, Genoa, Milan, Florence, Pisa and Rome could do no good."

In this condition he began "the use of ice, first melted in water, and then applied it in the solid cake to the person." At first he "took a sponge bath in a bowl of water, in which was dissolving a piece of ice the size of a walnut; from day to day larger lumps were used, and applied directly to his body, until finally he dissolved five or six pounds of ice upon his person every morning."

In a time not stated, the following changes occurred:—

"He gained sixty-five pounds of flesh, was restored not only to perfect health but to a state of vigorous energy, physical strength, vital power, unwasting glow of feeling and an ability to endure any amount of fatigue and exposure with apparent impunity. His description of his present condition is ravishing. Unbroken sleep, perfect control of his nervous system, mind always serene and cheerful, muscles firm and hard, no consciousness of the existence of his internal organs, ability to do with half the sleep he formerly required, appetite always good, digestion perfect, no taste whatever for unhealthy food; in short a supernatural state of mind and body, in which "every moment of his waking existence seems to be consciousness of physical, intellectual, moral and social happiness."

With the wisdom of his brother named above, he declares that to numerous pale, lean, sallow, dyspeptic, tobacco using, excess indulging authors, teachers, editors, clergymen, &c., this same remedy will bring unwonted power of mind and body, constant cheerfulness, a power of moral control, "a blessed clearness of thought," absence of all nervousness; in fine an ability to "walk further, stand up longer, work harder, and do everything better than he could do it before." "Existence will grow brighter, and the flame of life will burn with more calmness, serenity, glow and splendor than you ever dreamed of."

He attributes these wonderful transformations to the action of "certain chemical properties and the electrical heat which the ice contains," which explanation of the *modus operandi* of the matter is as philosophical and as lucid as could be given by an — ignoramus.

We would not advise the application of solid ice to old people or infants, or to any person of a frail constitution, without consulting a physician, for it would with great certainty hurry many to their graves. To have made the communication practically valuable, the writer should have stated the time it required to give him an increase of sixty-five pounds in weight; what he did in addition to the ice applications; what he did to place him in the deplorable condition described, and what bad practices he abandoned. Meanwhile, let the reader remember that applications or remedies which benefit one man may be reasonably expected to benefit another one, in proportion as the conditions of the two are alike, not merely in effect, but as to cause. No wise man would experiment on his own body and health and life on the loose statements of anonymous newspaper writers.

After all, when a reasonable allowance has been made for the evident exaggerations of the writer, there is not much that is unusual or remarkable in the changes. We have never known a man to gain sixty-five pounds in weight on ice, in a short time; but there are a good many who have "in the course of time" gained that much on vulgar beer. Salivated people have before now got well, by letting themselves alone; dyspeptic and lean folks, by simply ceasing to be pigs; and many a "bilious" man, as yellow as a pumpkin, has become as "hearty as a buck," by being simply compelled to go to work and make an honest living, which, by the way, is more health promoting, than the icebergs of a thousand poles. The trial will demonstrate this to almost any reader.

